# In memory of Hein Wellens: unique scientist, teacher, doctor and friend

**DOI:** 10.1007/s12471-020-01463-5

**Published:** 2020-07-13

**Authors:** A. P. M. Gorgels, P. A. Doevendans, A. A. M. Wilde

**Affiliations:** 1 Department of Cardiology, Maastricht University Medical Centre, Maastricht, The Netherlands; 2 Cardiologie Centra Nederland, Utrecht, The Netherlands; 3 University Medical Centre Utrecht, Utrecht University, Department of Cardiology, Utrecht, The Netherlands; 4 Central Military Hospital, Utrecht, The Netherlands; 5 Netherlands Heart Institute, Utrecht, The Netherlands; 6 Department of Clinical and Experimental Cardiology, Heart Center, Amsterdam UMC, University of Amsterdam, Amsterdam, The Netherlands

**Fig. 1:**
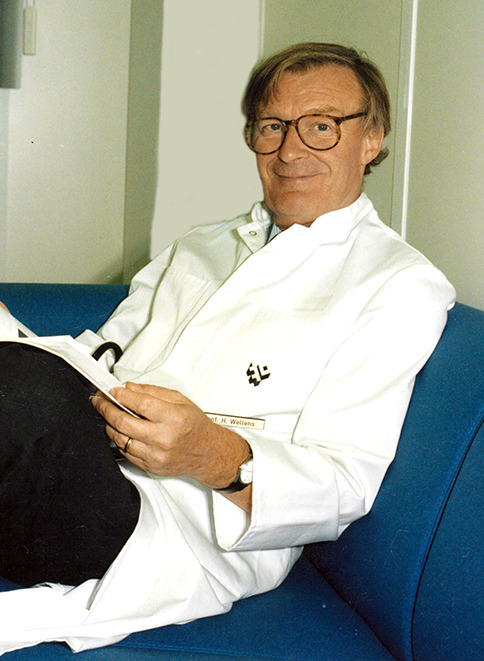
Professor Hein J.J. Wellens

Professor Henrick Joan Joost (Hein J.J.) Wellens (born 13 November 1935) (Fig. 1) died on Tuesday, 9 June 2020.

Hein Wellens started his scientific career in Amsterdam in the mid-1960s. His early research built on the pioneering work of Dirk Durrer in cardiac electrophysiology. He, Durrer and others were among the first to bring the pioneer experience in animal models to the clinic. His first paper was on the role of extrasystoles in the initiation and termination of supraventricular tachycardia in Wolff-Parkinson-White syndrome (1967). The results were predicted and the excitement was immense when the experiments confirmed what was anticipated. In the years thereafter similar breakthrough studies were performed in other supraventricular arrhythmias (atrial flutter, atrioventricular nodal re-entry). His thesis in 1971 summarised this work under the title ‘Electrical stimulation of the heart in the study and treatment of tachycardias’. The reproducible initiation and termination by pacing in ventricular arrhythmias was also demonstrated, enabling studies on the effect of antiarrhythmic drugs and providing the basis for new therapeutic approaches, including surgery. His inaugural lecture in 1974 was entitled *De extrasystole, vriend en vijand *(The extrasystole, friend and foe). He ended his lecture with the words: ‘I wanted to talk to you today about the profession that my love and libido has, cardiology’.

In 1977 he moved to Maastricht, where he was Chairman of the Department of Cardiology from 1 February 1977 until his retirement on 1 December 2000. His decision to start a new professional and social life in Maastricht, in the South Limburg area, was not an easy one and with unpredictable outcomes. At that time the area was in a state of flux owing to the recent closure of the coal mines. One of the measures of the central government to meet the need for new employment was to grant the area a medical faculty. Another argument for doing so was to introduce a novel teaching system, known as ‘problem-based learning’, which later proved to be a successful endeavour. The founding of this academic institution occurred, however, under suboptimal conditions, such as restricted budgeting, incorporation of the academic activities within the existing local clinical setting—thus without the perspective of a separate university hospital. Nevertheless, and typical for the attitude and confidence of Hein, he set up his department in early 1977 comprising a chairman, one secretary, one staff member, one fellow in training and with a single hospital bed. It would take until 1986 for the initial situation to be developed into a full-fledged academic hospital. In spite of these challenges Hein expeditiously started his clinical, teaching and scientific activities. Along with a training programme for residents in cardiology, an arrhythmia and electrophysiology fellowship programme was started, attracting many cardiology fellows from all over the world. This resulted in hundreds of publications in scientific books and journals, as well as PhD theses. With the continuation and expansion of the electrophysiology programme, conditions were created for definitive treatment of arrhythmias by surgical and catheter ablation. For this purpose, another important step forward was Hein’s achievement of receiving approval for a cardiothoracic surgery department.

Hein was passionate and dedicated to cardiology, which was his most important hobby. A unique gift was his ability to dismantle complex problems into simple steps to achieve an explanation. Many of us had the opportunity to attend teaching sessions on electrocardiography. His insight into the combination of electrical activity of the heart in relation to anatomy and haemodynamics was brilliant. Not only his skill in explaining it to us was exceptional. He was a strong supporter of maintaining the basic principles of cardiology as the starting point for clinicians and researchers. Every working day started with a meeting, attended by the full staff, fellows and residents, aiming not just to transfer patients, admitted during the night, but with lengthy discussions of patient cases with much emphasis on teaching and research. His teaching of residents could be very directive. This was based on the conviction that a cardiologist should be able to make crucial decisions in a split second, while at other times arrhythmias provided a puzzle that could always be resolved by reasoning and knowledge about the heart. His messages were underscored by imperatives such as: ‘Write it down, because you’ll find it nowhere in the books’, and ‘What you do not know, you do not recognise’, emphasising the need of clinicians for ready, solid knowledge. His basic motivation was his genuine interest in patients and their problem: ‘Listen to your patient’, he said frequently, and ‘It is nice to be important, but it is more important to be nice’. As such he demanded high professional standards not only from others but primarily from himself. His course on complex cardiac arrhythmias, which Hein gave together with his friend, the late Mark Josephson, for more than 30 years on both sides of the Atlantic, as well as their course on intracardiac tracings, attracted thousands of cardiologists from all over the world.

He had a clear vision of where the next step in cardiology would be made and directed and coached talented individuals to take up the new challenges to broaden the field of cardiology. Important was always the interaction with the technical contributors to the field (as in the early days in Amsterdam). He foresaw the key role that technology would play in medicine, but above all in cardiology. He realised that it would be crucial to bridge the gap between basic science and the clinic. Many cardiologists owe him gratitude for sharing his knowledge, creating opportunities and supporting people wherever possible. Through this attitude many patients profited from his work, often leading to a cure for arrhythmias and other cardiac problems.

Hein was intrigued by the problem of sudden cardiac arrest both from inside the hospital (how to identify the patient at risk) and from outside. He initiated a programme for a registry of cardiac arrest cases within the community, to install automated external defibrillators in public places, to organise citizen rescuers to act as first responders in cases of sudden cardiac arrest. An ultimate dream was to develop a device to detect ventricular fibrillation automatically.

Our thoughts and compassion are with Inez, who always strongly supported Hein’s academic and clinical aspirations, as well as with his children and grandchildren. In spite of his busy professional life Hein was always intensely involved with all the ups and downs of his family. We wish them much strength in this time of grief and refer to the last two lines of Horace Silver’s poem in bidding Hein farewell: ‘So when I am gone, I will have left a legacy of love’.

